# Follitropin delta combined with menotropin in patients at risk for poor ovarian response during in vitro fertilization cycles: a prospective controlled clinical study

**DOI:** 10.1186/s12958-023-01172-9

**Published:** 2024-01-02

**Authors:** Oscar Barbosa Duarte-Filho, Eduardo Hideki Miyadahira, Larissa Matsumoto, Lucas Yugo Shiguehara Yamakami, Renato Bussadori Tomioka, Sergio Podgaec

**Affiliations:** 1 Hospital Israelita Albert Einstein, São Paulo, Brazil; 2 Clínica VidaBemVinda, São Paulo, Brazil; 3 Disciplina de Obstetrícia e Ginecologia, Faculdade de Medicina, Universidade de São Paulo, São Paulo, Brazil

**Keywords:** In vitro fertilization, Controlled ovarian stimulation, Follitropin delta, Menotropin, Poor ovarian response

## Abstract

**Background:**

The maximum daily dose of follitropin delta for ovarian stimulation in the first in vitro fertilization cycle is 12 μg (180 IU), according to the algorithm developed by the manufacturer, and based on patient’s ovarian reserve and weight. This study aimed to assess whether 150 IU of menotropin combined with follitropin delta improves the response to stimulation in women with serum antimullerian hormone levels less than 2.1 ng/mL.

**Methods:**

This study involved a prospective intervention group of 44 women who received 12 μg of follitropin delta combined with 150 IU of menotropin from the beginning of stimulation and a retrospective control group of 297 women who received 12 μg of follitropin delta alone during the phase 3 study of this drug. The inclusion and exclusion criteria and other treatment and follow-up protocols in the two groups were similar. The pituitary suppression was achieved by administering a gonadotropin-releasing hormone (GnRH) antagonist. Ovulation triggering with human chorionic gonadotropin or GnRH agonist and the option of transferring fresh embryos or using freeze-all strategy were made according to the risk of developing ovarian hyperstimulation syndrome.

**Results:**

Women who received follitropin delta combined with menotropin had higher estradiol levels on trigger day (2150 pg/mL vs. 1373 pg/mL, *p* < 0.001), more blastocysts (3.1 vs. 2.4, *p* = 0.003) and more top-quality blastocysts (1.8 vs. 1.3, *p* = 0.017). No difference was observed in pregnancy, implantation, miscarriage, and live birth rates after the first embryo transfer. The incidence of ovarian hyperstimulation syndrome did not differ between the groups. However, preventive measures for the syndrome were more frequent in the group using both drugs than in the control group (13.6% vs. 0.6%, *p* < 0.001).

**Conclusions:**

In women with serum antimullerian hormone levels less than 2.1 ng/mL, the administration of 150 IU of menotropin combined with 12 μg of follitropin delta improved the ovarian response, making it a valid therapeutic option in situations where ovulation triggering with a GnRH agonist and freeze-all embryos strategy can be used routinely.

**Trial registration:**

U1111-1247-3260 (Brazilian Register of Clinical Trials, available at https://ensaiosclinicos.gov.br/rg/RBR-2kmyfm).

**Supplementary Information:**

The online version contains supplementary material available at 10.1186/s12958-023-01172-9.

## Background

Ovarian stimulation for in vitro fertilization (IVF) aims to produce multiple eggs and embryos. This allows embryo selection, and multiple transfers using the surplus embryos from a single ovarian stimulation, increasing the cumulative chances of pregnancy per initiated cycle [1]. The class of medication most commonly used for ovarian stimulation in IVF cycles are gonadotropins, [2] and their administration must adhere to the principles of efficacy and safety, which entails obtaining the number of eggs required to achieve viable embryos for transfer and avoiding excessive response, as seen in ovarian hyperstimulation syndrome (OHSS) [3].

In 2017, follitropin delta was approved for IVF, the first recombinant follicle-stimulating hormone (FSH) produced by a human cell line [4]. The dose of follitropin delta for ovarian stimulation is defined by an algorithm developed by the manufacturer based on patient’s weight and serum antimullerian hormone (AMH) levels. The algorithm aims to increase the chances of obtaining 8 to 14 eggs, the target response. This would help personalize treatment more than current therapeutic models, which rely solely on the doctor’s experience [5, 6]. In a Phase 3 study comparing the administration of follitropin delta in variable doses suggested by the algorithm with fixed-dose follitropin alpha in women undergoing their first IVF cycle, the group using follitropin delta had a higher frequency of target ovarian response and fewer extreme ovarian responses [7].

The algorithm has two limitations: first, in patients with AMH < 2.1 ng/mL, the maximum dose for first ovarian stimulation is 12 μg, equivalent to approximately 180 IU of FSH [8]. A previous systematic review showed that gonadotropin doses of 300–450 IU increase the number of oocytes retrieved in poor responders patients [9]. Second, the algorithm does not use luteinizing hormone (LH). According to several studies, the combined protocol using FSH plus LH may improve ovarian response during IVF cycles for patients at risk of low ovarian response [10–13].

It is clinically important to determine whether adding more FSH and LH to the dose of follitropin delta recommended by the manufacturer’s algorithm is effective and safe for increasing ovarian response in patients with AMH < 2.1 ng/mL when this new drug is used outside the manufacturer’s recommendations.

## Methods

### Aim, design, and setting of the study

The study aimed to assess whether combining 150 IU of menotropin, a gonadotropin of urinary origin that contains an equal proportion of FSH and LH, with the dose of follitropin delta determined by the algorithm, improves the ovarian response to IVF stimulation in patients with AMH < 2.1 ng/mL.

This is a prospective non-randomized controlled clinical study conducted in a single private human reproduction center in Sao Paulo, Brazil, to compare the use of follitropin delta associated with menotropin versus follitropin delta alone. The study was approved by the local ethics committee (Hospital Israelita Albert Einstein, CAAE19795519.4.0000.0071), registered in the Brazilian Registry of Clinical Trials (UTN code: 1111-1247-3260), and conducted in accordance with the norms and guidelines of the National Research Ethics Commission of the National Health Council of Brazil. All participants signed a written informed consent form before being included in the study.

### Characteristics of participants

This study included women aged 18–40 years with a serum AMH level < 2.1 ng/mL undergoing their first IVF cycle and diagnosed with unexplained infertility, tubal infertility, early-stage endometriosis or male infertility factors. The inclusion criteria also included body mass index between 17.5 and 32 kg/m^2^, regular menstrual cycles between 24 and 35 days, presence of both ovaries, and an early follicular phase serum FSH value between 1 and 15 IU/L. Advanced endometriosis, recurrent miscarriage, and hormonal therapy (excluding thyroid hormones) during the last menstrual cycle preceding the study were the main exclusion criteria. Additional Table 1 shows the entire inclusion and exclusion criteria.

### Controlled ovarian stimulation, in vitro procedures, and endometrial preparation

A historical and retrospective control group of 297 women with AMH < 2.1 ng/mL who participated in the Phase 3 study and received 12 μg of follitropin delta at a fixed daily dose based on the manufacturer’s algorithm (AMH < 2.1 ng/mL: 12 μg; AMH ≥2.1 ng/mL: 0.10–0.19 μg/kg; maximum daily dose of 12 μg) [7].

The prospective intervention group included 44 women who received subcutaneous 12 μg of follitropin delta (Rekovelle®, Ferring Pharmaceuticals) and 150 IU of menotropin (Menopur®, Ferring Pharmaceuticals), at a fixed daily dose during the stimulation.

The stimulations commenced on the 2nd or 3rd day of the menstrual cycle. On the 6th day of stimulation, both groups started the gonadotropin-releasing hormone (GnRH) antagonist, cetrorelix acetate at a dose of 0.25 mg/d (Cetrotide®, Merck Serono) throughout the trigger day. Triggering of final follicular maturation was done when three follicles or more reached ≥17 mm in average diameter. For women with < 25 follicles ≥12 mm, 250 μg of recombinant human chorionic gonadotropin (hCG) (Ovidrel®, Merck Serono) was administered. For those with 25–35 follicles ≥12 mm, 0.2 mg GnRH agonist triptorelin acetate (Gonapeptyl®, Ferring Pharmaceuticals) could be administered, or cancel the cycle. For women with > 35 follicles ≥12 mm, the cycle would have to be canceled. Poor follicular development, defined as the failure of three or more follicles to reach ≥17 mm in diameter after 20 days of stimulation, also resulted in cycle cancellation.

Oocyte collection took place 36 ± 2 hours after triggering final follicular maturation. The oocytes could be inseminated by IVF or intracytoplasmic sperm injection (ICSI) using ejaculated semen from the partner or a donor. For women triggered with agonists, all blastocysts were cryopreserved. For women in the control group receiving hCG, single blastocyst transfer was performed for women aged ≤37 years and ≥ 38 years with a blastocyst 3 BB or better; for all other situations, two blastocysts were transferred. In the intervention group, the same approach was suggested by the attending physician. However, the patient or couple made the final decision on the number of embryos to be transferred. Surplus embryos were cryopreserved for future use.

Vaginal micronized natural progesterone tablets (Utrogestan®, Besins Healthcare) 100 mg three times daily were administered for luteal phase support from the day after egg collection for 13–15 days and then discontinued when serum hCG confirmed pregnancy. Endometrial preparation was carried out for frozen embryo transfer cases, with 17-beta-estradiol transdermal gel 1.5 mg three times daily (Oestrogel®, Besins Healthcare). Ultrasound was performed at 5–6 weeks and 10–11 weeks after blastocyst transfer to confirm clinical and *ongoing* pregnancy, respectively. All pregnancies were followed up in person or remotely until 4 weeks after birth. Up to this point, adverse events have been recorded in accordance with informed consent.

### Comparisons and outcomes

The primary endpoint was the ovarian response obtained based on the following variables: number of eggs obtained; incidence of poor ovarian response according to the Bologna criteria of the European Society of Human Reproduction and Embryology (≤3 eggs retrieved or cycle canceled due to poor response) [14]; and estradiol levels on the day of the trigger.

Secondary outcomes included positive pregnancy test rate, clinical pregnancy rate (presence of gestational sac), implantation rate (number of gestational sacs/number of embryos transferred), ongoing implantation rate (number of embryos with heartbeat/number of embryos transferred), ongoing pregnancy rate (presence of heartbeat), miscarriage rate (gestational loss before 20 weeks), live birth rate (after fetal viability of 23 weeks), ovarian hypo-response (4–7 eggs), target ovarian response (8 –14 eggs) and high ovarian response (≥15 eggs), embryology endpoints (fertilization rate, quantity and quality of embryos) and adverse events. Safety outcomes included the proportion of women with early and late OHSS, including moderate and severe grades, according to the Golan classification [15] and preventive interventions for early OHSS (i.e., cycle cancellation, trigger with GnRH agonist, freezing all embryos for deferred transfer or dopaminergic agonist in women with ≥20 follicles of ≥12 mm).

### Statistical analysis

The sample was characterized using the mean and standard deviation, minimum and maximum, median and quartiles for quantitative variables, and absolute and relative frequencies for qualitative variables [16].

Comparisons between groups were checked using Mann–Whitney tests for quantitative outcomes and chi-square or Fisher’s exact tests for qualitative outcomes. Data normality was checked using the Shapiro–Wilk test, box plot graphs, histograms and quantile comparison graphs. The analyses were carried out using R and the Statistical Package for the Social Sciences, v.26.0, with a significance level of 5% [16–19].

Study data were collected and managed using REDCap electronic data capture tools hosted at Hospital Israelita Albert Einstein.

### Power calculation

We defined obtaining three more eggs in the intervention group as a clinically relevant result, as it would be sufficient to obtain one more embryo and increase the chances of pregnancy. To this end, we used as a reference an average fertilization rate per injected egg of 75% and a blastulation rate per formed zygote of 50%, based on the Vienna Consensus on the performance of IVF laboratories and the results of our center [20]. Considering the average number of eggs obtained in the phase 3 study for this group of women with AMH < 2.1 ng/mL of 7.54 (standard deviation 4.57) and an ovarian response with three more eggs, we calculated that 44 patients would be enough to demonstrate a statistically significant difference in favor of the intervention, with a power of 90% and an alpha error of 5%. (NCSS L. PASS 14 Power Analysis and Sample Size Software. 2015. ncss.com/software/pass).

## Results

### Characteristics of the group included in the study

IVF cycles in the intervention arm were performed between May 2020 and September 2021, with live birth follow-up completed in July 2022. We screened 97 consecutive patients with prior AMH < 2.1 ng/mL for enrollment until the 44 eligible patients were completed. We excluded 53 patients/couples after initial assessment: two had irregular menstrual cycles at the time of study initiation, two did not have a diagnosis of infertility, three had already undergone previous IVF cycles, four had a body mass index above 32 kg/m^2^ at the time of the cycle start, four had repeated AMH by the electrochemiluminescence method and the result was > 2.1 ng/mL, three had baseline FSH > 15 IU/L, 15 had a positive serology/test for an infectious disease (including Covid-19 and Zika virus), three had a diagnosis of advanced endometriosis, two had a karyotype alteration in one of the members of the couple, two had a sperm concentration of < 1 million sperm in the month prior to the start of stimulation, eight had a systemic clinical disease detected as poorly controlled at the time of initial screening (diabetes, hypertension, autoimmune disease), two became pregnant immediately before starting treatment and three did not complete screening in time to be included in the study.

### Demographic and baseline characteristics

Table 1 shows the demographics and baseline characteristics of the 44 patients in the intervention group and the 297 in the control group. No significant difference was observed between the groups regarding age and AMH levels. Patients in the intervention group were heavier and had lower antral follicle counts, and women in the intervention group had significantly lower baseline FSH levels. The groups also differed in ethnicity, infertility length, etiology, and baseline TSH and progesterone levels.

**Table 1:** Baseline demographic characteristics

	Folitropin Delta + Menotropin (*n* = 44)	Folitropin Delta (*n* = 297)	*p*-value
Age (years)			
All women	35.8 ± 3.4	34.3 ± 3.70	0.051^c^
Age distribuition			
<35	15 (34%)	147 (49%)	0.120^a^
35-37	14 (32%)	83 (28%)	
38-40	15 (34%)	67 (23%)	
Ethnicity			**< 0.001**^**b**^
White	26 (59.1%)	287 (96.6%)	
Brown or black	16 (36.4%)	2 (0.7%)	
Yellow	2 (4.5%)	8 (2.7%)	
Weight (kg)	70.4 ± 11.5	64.9 ± 10.2	**< 0.001**^**c**^
BMI (Kg/m^2^)	25.9 ± 3.2	23.7 ± 3.35	**< 0.001**^**c**^
Time of infertility (in months)	58.2 ± 38.2	37.2 ± 27.2	**< 0.001**^**c**^
Cause			**< 0.001**^**b**^
Tubal abnormalities	19 (52.8%)	41 (13.8%)	
Unexplained	8 (18.2%)	125 (42.1%)	
Endometriosis stage I/II	5 (13.9%)	11 (3.7%)	
Male factor	16 (44.4%)	118 (39.7%)	
Other	0 (0)	2 (0.7%)	
Ovarian reserve markers			
AFC, 2 -10 mm (n)	9.5 ± 3.89	11.5 ± 5.06	**0.007**^**c**^
AMH (ng/mL)	1.20 ± 0.50	1.18 ± 0.50	0.495^c^
Hormonal profile			
Basal FSH (IU/L)	7.1 [5.9 - 8.9]	8.5 [6.9 - 10.6]	**0.003**^**c**^
Basal estradiol (pg/mL)	49.9 [36.1 - 65.9]	43.6 [33.2- 55.0]	**0.024**^**c**^
Progesterone (ng/mL)	0.8 [0.6 - 1.2]	0.5 [0.2 - 0.7]	**< 0.001**^**c**^
TSH (mIU/L)	1.8 [1.3 - 2.4]	1.5 [1.1 - 2.0]	**0.005**^**c**^

^a^Chi-square test

^b^Fisher’s exact test

^c^Mann-Whitney test

Mean ± SD; median [IQR]

### Ovarian response and embryology outcomes

Table 2 shows the results related to the response to ovarian stimulation and the embryological and laboratory outcomes. The duration of stimulation was significantly longer in the intervention group: 1.26 days longer, which resulted in prolonged administration of follitropin delta. However, the daily dose was the same (12 μg) in both groups. Estradiol levels on the trigger day were also significantly higher in this group. The intervention group had significantly higher fertilization rates, more zygotes, blastocysts, and top-quality blastocysts. Although intervention group had 1.53 more retrieved eggs, fewer poor and hypo ovarian responses and more target and high ovarian responses than the control group, these differences were not statistically significant.

**Table 2:** Ovarian response, embryology, and safety outcomes

	Folitropin Delta + Menotropin (*n* = 44)	Folitropin Delta (*n* = 297)	*p*-value
Ovarian response outcomes			
Duration of stimulation (days)	9.95 ± 1.48	8.69 ± 1.63	**< 0.001**^**c**^
Total dose of follitropin delta (μg)	119.4 ± 17.7	104 ± 20.2	**< 0.001**^**c**^
Total dose of menotropin	1492.5 ± 222.0	0	–
Cancelled due to poor response	2 (4.5%)	14 (4.7%)	> 0.999^b^
Estradiol on trigger day (pg/mL)	2150.2 ± 1093.3	1372.8 ± 741.7	**< 0.001**^**c**^
Oocytes retrieved	9.07 ± 5.56	7.54 ± 4.57	0.058^c^
Ovarian response			0.276^b^
< 4 eggs	6 (13.6%)	47 (16%)	
4 - 7 eggs	16 (36.4%)	118 (40.1%)	
8 - 14 eggs	16 (36.4%)	110 (37.4%)	
≥ 15 eggs	6 (13.6%)	19 (6.5%)	
Embryology outcomes			
Zygotes (2 PN)	5.1 (3.05)	4.2 (3.10)	**0.040**^c^
Fertilization rate	85.9 ± 15.8	56.7 ± 25.4	**< 0.001**^c^
Blastocysts (total)	3.10 ± 2.66	2.42 ± 2.22	**0.030**^c^
Blastocysts (top quality)	1.80 ± 2.01	1.37 ± 1.71	**0.017**^**c**^
Blastocysts transferred (fresh or thawed)			**< 0.001**^**b**^
*No transfer*	7 (15.9%)	50 (16.84%)	
1	26 (59.1%)	232 (78.11%)	
2	11 (25%)	15 (5.05%)	
Cryopreserved surplus blastocysts	1.00 ± 1.53	1.03 ± 1.17	0.486^c^
Individuals with frozen embryos	26 (61.9%)	154 (51.9%)	0.291^a^
Safety outcomes			
Preventive measures for OHSS (GnRHa trigger, freeze-all)	6 (13.6%)	2 (0.6%)	**< 0.001**^**b**^
Early OHSS	2 (4.5%)	2 (0.7%)	0.082^b^
OHSS any degree	2 (4.5%)	4 (1.3%)	0.174^b^

^a^Chi-square test

^b^Fisher’s exact test

^c^Mann-Whitney testMean ± SD

Two cycles were cancelled due to low response during ovarian stimulation, both in patients with an AMH < 0.5 ng/mL. A third patient in this group did not have any eggs retrieved during collection. Of the five patients with AMH < 0.5 ng/mL, only two had an embryo transfer.

The trend towards a higher frequency of high ovarian response in the menotropin group led to a higher incidence of preventive interventions for OHSS. All six patients with ≥15 eggs retrieved had AMH > 1.0 ng/mL and all three patients with ≥20 eggs had AMH > 1.5 ng/mL.

No statistically significant difference was observed in the incidence of OHSS. The two cases in the intervention group were mild and early (up to 9 days after triggering in patients who froze all the embryos).

### Pregnancy outcomes

Table 3 shows the clinical results. No statistically significant difference was observed between the intervention group and the control group regarding the positive pregnancy test rate, clinical pregnancy rate, ongoing pregnancy rate, miscarriage rate, implantation rate, ongoing implantation rate, and live births. In the intervention group, where two embryos were transferred more often, twin pregnancies were higher.

**Table 3:** Pregnancy outcomes

	Folitropin Delta + Menotropin (*n* = 44)	Folitropin Delta (*n* = 297)	*p*-value
Outcomes per cycle started			
hCG positive	17 (38.6%)	109 (36.7%)	0.361^a^
Clinical pregnancy	15 (34.1%)	98 (33.0%)	0.465^a^
Ongoing pregnancy	12 (27.3%)	84 (28.3%)	0.739^a^
Miscarriage	5 (29.4%)	25 (22.9%)	0.550^b^
Implantation rate	39.6%	37.4%	0.901^a^
Ongoing implantation rate	29.2%	32.1%	0.820^a^
Live birth	12 (27.3%)	82 (27.6%)	> 0.999^a^
Twin pregnancy	2 (16.7%)	0	**0.014**^**b**^

^a^Chi-square test

^b^Fisher’s exact test

We included only the results of the first embryo transfer to make the comparisons, because cumulative results were not available for the historical control group. The results of the transfers of the surplus frozen embryos are listed in the Additional table 2.

## Discussion

This study evaluated the effects of combining 150 IU of menotropin with follitropin delta on ovarian response during IVF stimulation in patients with serum AMH < 2.1 ng/mL. The administration of follitropin delta combined with menotropin has been reported in controlled analyses [21] and uncontrolled real-life studies [22]. However, to the best of our knowledge, this is the first controlled study to test follitropin delta combined with menotropin at these doses for this specific group of patients. Serum estradiol levels were significantly higher in the intervention group (2150 pg/mL vs. 1372 pg/mL, *p* < 0.001), indicating a higher ovarian response with menotropin combined with follitropin delta compared to the administration of follitropin delta alone. Furthermore, although the higher number of eggs in the study group did not reach statistical significance, this group had significantly more blastocysts and more good quality blastocysts to transfer, which, in the end, is more important to IVF success.

We used phase 1 and 2 studies with follitropin delta to develop this study, estimating that 12 μg of the drug would be equivalent to 150–200 IU [23–25]. This inference was confirmed a posteriori *in* a dose equivalence study published in 2020, which showed that 10 μg of follitropin delta is equivalent to approximately 150 IU of FSH [8]. Therefore, the maximum dose of follitropin delta for a first IVF cycle determined by the algorithm, 12 μg, is equivalent to approximately 180 IU of FSH daily, which we consider to be an underdose for patients at risk of low response. A systematic review from Cochrane Library, last updated in 2018, reported that using doses of 300–450 IU of recombinant FSH instead of 150 IU resulted in a higher number of eggs for poor responders [9]. Thus, it seems very plausible from a biological point of view that the improvement in ovarian response we observed may have occurred due to the addition of 150 IU of FSH activity for this group of patients, amounting to approximately 300–350 IU of FSH daily in the intervention group compared to 180 IU in the control group.

We believe that a group of patients with a very low ovarian reserve could benefit from using lower gonadotropins doses, so-called minimal and mild stimulation, or even modified natural and natural cycles extensively discussed in the literature under a well-known “less is more” concept [26–29]. In our study, 60% of women with AMH < 0.5 ng/mL had their cycle canceled due to lack of follicular growth, absence of mature eggs or embryos available for transfer. Despite being a small subgroup of five patients, we did not rule out the possibility they could benefit from low doses of gonadotropins stimulation or natural cycle if they choose to use their own eggs for IVF. This strategy can reduce treatment burden, side effects, and costs. The hypothesis that this very low ovarian reserve patients do not benefit by increasing gonadotropins dose was obviously not tested in this study, but future research could examine it.

A study by Bosch et al. published in 2019 reported that using higher doses of follitropin delta in the second and third IVF cycles (average daily doses of 12.0 ± 3.6 μg and 14.6 ± 5.1 μg, respectively) improved ovarian response in patients with low response in the first cycle, clearly indicating that there is room to administer doses > 12 μg of follitropin delta (or 180 IU of FSH) in patients at risk of low response [30]. We could simply have given higher doses of follitropin delta to patients with AMH < 2.1 ng/mL. However, we believe that the benefit of combining menotropin for ovarian response is not only in function of FSH activity but also because this drug contains LH acitivity, which would be an advantage over merely increasing the dose of follitropin delta. The addition of LH to FSH has been shown to improve ovarian response in women with low ovarian reserve [13, 31] by a mechanism that seems to involve an LH-induced increase in FSH responsiveness [32].

Our study also found improved fertilization rates, blastocysts number, and top-quality blastocysts number in the menotropin group which may have been a result of the addition of LH activity to the stimulation protocol. A Canadian study by Bissonnete et al., published in 2021, found that follitropin delta combined with menotropin increased blastocyst number and quality [21]. That study, unlike ours, included patients from the general population with no restrictions on AMH levels. In addition, the dose of menotropin was variable according to the dose of follitropin delta and the patient’s weight. Previous studies have shown a qualitative benefit of adding LH to the ovarian response for patients with low ovarian reserve (AMH < 1.2 ng/mL), and in those > 35 years of age [11, 12, 31]. In both, the study by Bissonnete et al. and ours, the improvement in the quantity and quality of blastocysts obtained may have been due to the addition of the LH contained in menotropin. In the Canadian study, 50% of the patients were > 35 years old. In our study, 47 and 54% of the women had AMH < 1.2 ng/mL and were over 35 years old, respectively. Although this beneficial effect of LH is plausible, a difference in performance between the IVF laboratories in the multicenter phase 3 study and the one in this study, as well as the use of ICSI in 100% of cases in the intervention group cannot be ruled out as the cause of these better fertilization rate, blastulation rate and better blastocyst quality observed.

Regarding ovarian response, we found a statistically significant increase in measures to prevent hyperstimulation, in the intervention group, such as GnRHa trigger and freeze-all. Administering a higher dose of gonadotropins, a known risk factor for high ovarian responses and OHSS, seems to have caused this response [33–35]. All the cases with a high (> 14 eggs) or very high (≥20 eggs) ovarian response had AMH levels > 1.0 ng/mL and ≥ 1.5 ng/mL, respectively. So, consider adding lower daily doses of menotropin, such as 112.5 IU or 75 IU, to patients with AMH levels between 1.0 and 2.1 ng/mL, may be a good alternative in a scenario where fresh embryo transfer is a priority. In our country, however, the use of GnRHa trigger and freeze-all are frequent. The freezing of all embryos is routine, not only for the prevention of OHSS, but also to allow preimplantation genetic test as well as a strategy to increase endometrial receptivity and reduce obstetric complications, as previously demonstrated in the literature [36–38]. Therefore, in the trade-off between having a fresh transfer or the highest possible number of eggs, the latter is more desirable in our daily practice. The intervention group had more blastocysts (3.10 vs. 2.42, *p* = 0.030) and better-quality blastocysts (1.80 vs. 1.37, *p* = 0.017) available for transfer, but this fact did not increase pregnancy rates after the first embryo transfer. However, we cannot rule out a long-term change in cumulative pregnancy rates after two or more embryo transfers.

Our study was not designed to demonstrate possible differences in pregnancy, implantation, miscarriage and live birth outcomes. The only gestational outcome with a difference was twin pregnancies (16.7% vs. 0, *p* = 0.014), due to the greater number of couples who opted for double embryo transfer in the intervention group (25% vs. 5.05%, *p* < 0.001). We understand those who advocate the use of a single live birth at term as the ideal primary outcome from the patient’s point of view in assisted reproduction studies [39, 40], but this does not seem to us to be the appropriate or even feasible endpoint for studies of ovarian stimulation interventions. There are plausible arguments for this: (i) between ovarian stimulation and the birth of the baby there are many variables that in no way depend on stimulation, such as seminal quality, IVF laboratory conditions, the couple’s intention to transfer two embryos or do PGT and even prenatal care [41]; (ii) the number of individuals to be allocated per group to demonstrate differences in the live-birth outcome in a low-reserve population (e.g., AFC < 10) can be as high as 2000 [41]. The multicenter phase 3 trial that served as the control group for this study, for example, had 297 patients with AMH < 2.1 ng/mL. Since robust evidence indicates that retrieving more eggs after ovarian stimulation increases pregnancy rates, we believe that using ovarian response endpoints, such as the number of eggs is a valid and appropriate strategy to avoid these difficulties. Sunkara et al. studied more than 400,000 IVF cycles with fresh embryo transfers and found that the more eggs in the range between 1 and 15 were obtained, regardless of age, the greater the chances of a live birth [42]. According to the findings of Law et al., with > 220,000 IVF cycles, and Polyzos et al., with approximately 15,000 IVF cycles, considering all the embryo transfers from the same stimulation and not just the fresh transfer, the more eggs were obtained, regardless of the number, the greater the chances of having a live birth, in all the age groups [43, 44].

Assessing the cost-effectiveness of any medical intervention is a crucial point to discuss. Considering Brazilian scenario, we estimated that the additional cost per patient (medication plus endometrial preparation due to the freeze-all strategy) was approximately 1167 euros in the intervention group. If we consider that cases with surplus frozen embryos were more frequent in the intervention group (61.9% versus 51.9% in control arm), there could be a cost reduction of around 282 euros per patient due to IVF cycles that would not be needed in case of not reaching the pregnancy after the first transfer. Consequently, the inclusion of only direct costs would have resulted in an additional expenditure of approximately 885 euros per patient. However, one cannot ignore the great physical and emotional burden of carrying out a new IVF cycle for patients who did not become pregnant after the first cycle or who planned to have more than one child and did not have surplus embryos, a situation that was more prevalent in the control group (48.1% versus 38.1% in the intervention arm). The last scenario, involving family planning for an additional child, could lead to future savings in the intervention group. However, calculating these savings is challenging, given the ovarian aging that will occur in 2 years or more. When these patients, who are currently 35 years old, return for a new treatment, the absence of frozen embryos for use adds to the complexity of the calculation.

A limitation of our study is that it is not randomized, with a retrospective historical control group. Although we used the same inclusion and exclusion criteria as the phase 3 study from which the control group originated, the intervention group had a lower antral follicle count than the control group (9.5 vs. 11.5, *p* = 0.007), which is important for a study on ovarian stimulation and response. Thereby one way we found of improving the comparison between the responses was to use indices that relativized the number of eggs obtained as a function of the initial antral follicles. One index, for example, suggested by Alviggi et al., is the *Follicle-to-oocyte index* (FOI = number of mature eggs × 100/number of antral follicles), with a FOI > 50% being considered normal [45]. Our control group, which used 12 μg of follitropin delta, had an average FOI of 66%, while the intervention group, using the combination of follitropin delta and menotropin, had an average FOI of 94%, a statistically significant difference (*p* < 0.001), which reinforces the positive effect of the intervention on the ovarian response. It should be remembered that this and all the other comparisons in this study were carried out with grouped results and not paired on a case-by-case basis due to the limited access to individual data from the phase 3 study for ethical reasons.

## Conclusions

The administration of menotropin combined with follitropin delta enhanced ovarian stimulation in IVF cycles in patients with AMH < 2.1 ng/mL resulting in higher serum estradiol levels, more and better-quality blastocysts compared to administration of follitropin delta alone at the manufacturer’s recommended dose. No difference in pregnancy rates after the first embryo transfer was observed between the groups. No severe OHSS cases occurred, although interventions for preventing the syndrome were needed more frequently. Therefore, the combination tested in this study is a valid therapeutic option to improve ovarian response in settings where GnRH agonist triggering and freeze-all embryos could be routinely used.

### Supplementary Information

**Additional file 1: Additional Table 1.** Inclusion and exclusion criteria. **Additional Table 2.** Outcomes after subsequent embryo transfers (updated in November 2023).

## Data Availability

Study data were collected and managed using REDCap electronic data capture tools hosted at Hospital Israelita Albert Einstein. These datasets used and/or analysed during the current study are available from the corresponding author on reasonable request. The control group dataset is available from Ferring Pharmaceuticals but restrictions apply to the availability of these data, which were used under license for the current study, and so are not publicly available. Data are however available from the authors upon reasonable request and with permission of Ferring Pharmaceuticals.

## Abbreviations

AMH: Antimullerian hormone
FSH: Follicle stimulating hormone
GnRH: Gonadotropin releasing hormone
hCG: Human chorionic gonadotropin
IVF: In vitro fertilization
ICSI: Intracytoplasmic sperm injection
LH: Luteinizing hormone
OHSS: Ovarian hyperstimulation syndrome
