# Supplementary material for: Follitropin delta combined with menotropin in patients at risk for poor ovarian response during in vitro fertilization cycles: a prospective controlled clinical study
Source: Reprod Biol Endocrinol. 2024 Jan 2;22:7. doi: 10.1186/s12958-023-01172-9 (PMC10759374; doi:10.1186/s12958-023-01172-9)
Supplement: Supplementary file 1 — Additional file 1: Additional Table 1. Inclusion and exclusion criteria. Additional Table 2. Outcomes after subsequent embryo transfers (updated in November 2023). [file 12958_2023_1172_MOESM1_ESM.docx]

**Additional table 1: Inclusion and exclusion criteria**

|  |
| --- |
| **Inclusion criteria** |
| 1. Signed informed consent before the screening evaluations |
| 2. Good physical and mental health |
| 3. Pre-menopausal women between the ages of 18 and 40. At least 18 years old at the time of signing the consent form and no more than 40 years old at the start of stimulation |
| 4. Infertility diagnosed by tubal factor, unexplained infertility, stage I/II endometriosis, male factor, eligible for IVF or ICSI with ejaculated semen or semen bank |
| 5. Infertility for at least 1 year prior to screening for women ≤ 37 years or 6 months for women ≥ 38 years (except severe tubal or male factor) |
| 6. The study cycle was the first ovarian stimulation cycle for IVF/ICSI |
| 7. Regular menstrual cycles between 24 and 35 days, presumed to be ovulatory |
| 8.Hysterosalpingography, hysteroscopy, hysterosonography, or transvaginal ultrasound documenting a uterus consistent with pregnancy (i.e., no evidence of submucosal or intramural fibroids >3 cm in diameter, no polyps, no congenital abnormalities that are associated with a reduced chance of pregnancy) within one year of starting the study. |
| 9. Ultrasound documenting the presence of both ovaries, with no evidence of significant abnormalities (endometrioma >3 cm or enlarged ovaries that contraindicate the use of gonadotropins) and normal appendages (no hydrosalpinx) within one year of the start of the study. |
| 10. Serum FSH levels (between days 2 and 4 of the cycle) between 1 and 15 IU/L, results obtained within 3 months of the start of the study. |
| 11. Negative serologies for hepatitis B, hepatitis C, HIV, primate T-lymphotropic virus, syphilis, and Zika virus within 6 months of egg collection |
| 12. Body mass index between 17.5 and 32 kg/m^2^ at the time of screening |
| 13. Agree to transfer preferably one embryo and no more than two embryos, regardless of age, for fresh cycles |
| 14. Agree to transfer preferably one embryo and no more than two embryos, regardless of age, for the first frozen cycle within 3 years of egg collection |
| 15. Serum AMH between 0.1 and 2.1 ng/mL, results obtained within 6 months of the start of the study |
| **Exclusion criteria** |
| 1. Stage III/IV endometriosis |
| 2. One or more follicles ≥10 mm observed on transvaginal ultrasound at the start of stimulation |
| 3. Known history of recurrent miscarriage (3 or more consecutive miscarriages confirmed by ultrasound before 24 weeks of gestation, except ectopic pregnancy |
| 4. Known karyotype abnormality. If the sperm concentration is <1 million/mL, karyotyping and Y chromosome microdeletion research are mandatory. |
| 5. Any significant clinical systemic disease |
| 6. Hereditary or acquired thrombophilia |
| 7. Arterial or venous thromboembolism or severe thrombophlebitis, or a history of these events |
| 8. Known porphyria |
| 9. Any endocrine or metabolic disease (pituitary, adrenal, pancreas, liver, or kidney) that compromises participation in the study, except well-controlled thyroid disease |
| 10. Known presence of anti-FSH antibodies |
| 11. Known tumors in the breast, ovary, adrenal, pituitary, or hypothalamus) that contraindicate the use of gonadotropins |
| 12. Known moderate or severe impairment of hepatic or renal function |
| 13. Current breastfeeding |
| 14. Vaginal bleeding without diagnosis |
| 15. Cervical cytology abnormality of clinical significance not resolved within 3 years of the start of the study |
| 16. Gynecological examination findings during screening that contraindicate the use of gonadotropins or contraindicate pregnancy (congenital anomalies of the uterus or retained intrauterine device) |
| 17. Pregnancy (positive urine test performed on the day the study begins) or contraindication to pregnancy |
| 18. Active pelvic inflammatory disease |
| 19. Use of fertility modifiers in the cycle prior to treatment with dehydroepiandrosterone, contraceptives, progestogens or estrogens |
| 20. Use of hormonal preparations (except thyroid hormones) in the month prior to the study |
| 21. Previous history of chemotherapy (except for gestational conditions) or radiotherapy |
| 22. Drug or alcohol abuse in the year prior to the study or drinking more than 14 units of alcohol per week in the month prior to the study |
| 23. Smoking more than ten cigarettes/day in the 3 months prior to the study |
| 24. Known hypersensitivity to the drugs to be used in the study |
| 25. Participation in previous studies |
| 26. Use of any unregistered experimental drug in the 3 months prior to the start of this study |

**Additional table 2: Outcomes after subsequent embryo transfers (updated in November 2023)**

| ID | Number of embryo transfers | Date of second embryo transfer | Number of blastocysts transferred | Number of top-quality blastocysts transferred | hCG test | Gestational sac | Cardiac activity | Pregnancy outcome | Date of third embryo transfer | Number of blastocysts transferred | Number of top-quality blastocysts transferred | hCG test | Gestational sac | Cardiac activity | Pregnancy outcome |
| --- | --- | --- | --- | --- | --- | --- | --- | --- | --- | --- | --- | --- | --- | --- | --- |
|  |  |  |  |  |  |  |  |  |  |  |  |  |  |  |  |
| 2 | 3 | 11/17/2020 | 2 | 2 | Positive | No | - | Biochemical | 05/06/2021 | 2 | 2 | 1 | 1 | 1 | Live birth |
| 6 | 2 | 09/16/2022 | 1 | 1 | 0 | - | - | Negative | - | - | - | - | - | - | - |
| 18 | 3 | 01/02/2021 | 2 | 2 | 0 | - | - | Negative | 04/08/2021 | 2 | 0 | 1 | 0 | - | Biochemical |
| 20 | 2 | 11/27/2020 | 1 | 1 | Positive | No | - | Biochemical | - | - | - | - | - | - | - |
| 21 | 2 | 04/14/2023 | 2 | 2 | Positive | Yes | Yes | Ongoing | - | - | - | - | - | - | - |
| 24 | 2 | 02/21/2022 | 2 | 2 | Positive | Yes | Yes | Live birth | - | - | - | - | - | - | - |
| 36 | 2 | 08/28/2021 | 1 | 1 | 0 | - | - | Negative | - | - | - | - | - | - | - |
| 82 | 2 | 13/08/2022 | 1 | 1 | Positive | Yes (2) | Yes (2) | Miscarriage | - | - | - | - | - | - | - |
| 97 | 2 | 09/28/2023 | 1 | 1 | Positive | Yes | Yes | Ongoing | - | - | - | - | - | - | - |
